# Light-Emitting Diodes and Liquid System Affect the Caffeoylquinic Acid Derivative and Flavonoid Production and Shoot Growth of *Rhaponticum carthamoides* (Willd.) Iljin

**DOI:** 10.3390/molecules29092145

**Published:** 2024-05-05

**Authors:** Ewa Skała, Monika A. Olszewska, Przemysław Tabaka, Agnieszka Kicel

**Affiliations:** 1 Department of Biology and Pharmaceutical Botany, Medical University of Lodz, Muszynskiego 1, 90-151 Lodz, Poland; 2 Department of Pharmacognosy, Medical University of Lodz, Muszynskiego 1, 90-151 Lodz, Poland; monika.olszewska@umed.lodz.pl (M.A.O.); agnieszka.kicel@umed.lodz.pl (A.K.); 3 Institute of Electrical Power Engineering, Lodz University of Technology, 90-537 Lodz, Poland; przemyslaw.tabaka@p.lodz.pl

**Keywords:** liquid medium, LED, caffeoylquinic acid derivatives

## Abstract

Plant in vitro cultures can be an effective tool in obtaining desired specialized metabolites. The purpose of this study was to evaluate the effect of light-emitting diodes (LEDs) on phenolic compounds in *Rhaponticum carthamoides* shoots cultured in vitro. *R. carthamoides* is an endemic and medicinal plant at risk of extinction due to the massive harvesting of its roots and rhizomes from the natural environment. The shoots were cultured on an agar-solidified and liquid-agitated Murashige and Skoog’s medium supplemented with 0.1 mg/L of indole-3-acetic acid (IAA) and 0.5 mg/L of 6-benzyladenine (BA). The effect of the medium and different treatments of LED lights (blue (BL), red (RL), white (WL), and a combination of red and blue (R:BL; 7:3)) on *R. carthamoides* shoot growth and its biosynthetic potential was observed. Medium type and the duration of LED light exposure did not affect the proliferation rate of shoots, but they altered the shoot morphology and specialized metabolite accumulation. The liquid medium and BL light were the most beneficial for the caffeoylquinic acid derivatives (CQAs) production, shoot growth, and biomass increment. The liquid medium and BL light enhanced the content of the sum of all identified CQAs (6 mg/g DW) about three-fold compared to WL light and control, fluorescent lamps. HPLC-UV analysis confirmed that chlorogenic acid (5-CQA) was the primary compound in shoot extracts regardless of the type of culture and the light conditions (1.19–3.25 mg/g DW), with the highest level under R:BL light. BL and RL lights were equally effective. The abundant component was also 3,5-di-*O*-caffeoylquinic acid, accompanied by 4,5-di-*O*-caffeoylquinic acid, a tentatively identified dicaffeoylquinic acid derivative, and a tricaffeoylquinic acid derivative 2, the contents of which depended on the LED light conditions.

## 1. Introduction

*Rhaponticum carthamoides* (Willd.) Iljin. is an endemic plant species occurring in the natural habitats in Siberia (in the Altai and Sayan Mountains), Mongolia, Kazakhstan, and China (mainly in the Heilongjiang Province) [1,2,3,4]. This species has been used in the traditional medicine of Siberia for treating physical fatigue and weakness after illness [1]. The monograph of the raw material—the roots and rhizomes (*Rhapontici carthamoidis rhizomata cum radicibus*) is included in official pharmacopoeias of some Eastern European countries. This raw material is classified as a tonic and adaptogen agent [3]. *R. carthamoides* is an endangered species due to the excessive grazing of animals and over-exploitation of raw material for medicinal purposes [5,6] and is monographed in the Red List of the Russian Federation and regional Red List [5]. In recent years, the intensification of field cultivation of medicinal plants has been observed, but many species are still harvested from the natural habitat, which results in a gradual shrinking of natural resources. In addition, conventional plant propagation is very slow and inefficient for many species. Furthermore, the acquisition of specialized metabolites from plants is limited due to their low content. An alternative method may be in vitro micropropagation of plants. This method may also be profitable for protecting rare and endangered plant species threatened with extinction and for the cryopreservation of genetic materials or propagation of medicinal and economically significant plants all year round [7]. In addition, micropropagation allows the obtaining of lines with the desired morphological characteristic or that are highly productive in terms of specific specialized metabolites. The plant materials obtained using biotechnological methods are high quality and genetically homogeneous. Basal medium composition, growth regulators, or medium consistency are known agents that regulate shoot growth. In addition, the light quality, spectrum, intensity, or photoperiod are also essential factors that can influence shoot growth, plant quality, plant biomass, and secondary metabolism [8,9,10].

*R. carthamoides* is a species intensively harvested from its natural habitat. Therefore, its natural states are threatened with extinction. This plant species has not been intensively studied in vitro by researchers. Previously, *R. carthamoides* shoots were obtained through direct and indirect somatic organogenesis [11,12,13]. Our former studies described the protocol of *R. carthamoides* transformation using *Rhizobium rhizogenes* [14]. In addition, the pRi-transformed shoots and plants were obtained [15]. *R. carthamoides* in vitro cultures were found to be an efficient source of CQAs [13,14,15,16].

The current study focuses on the influence of light-emitting diode (LED) treatment with different wavelengths (along with the fluorescent lamps as the control) and the type of culture (an agar-solidified and liquid-agitated) on the shoot growth and propagation of *R. carthamoides* from the fragments of the stem with axillary bud. The study also optimized the best condition for high-yielding *R. carthamoides* shoot cultures with an effective production of phenolic specialized metabolites, i.e., caffeoylquinic acid derivatives and flavonoids. No previous reports describe the effect of LED illumination and the liquid system on the propagation of *R. carthamoides* shoots and the biosynthesis of their specialized metabolites. However, numerous papers documented the positive effect of LEDs on in vitro culture growth of other plant species and their biosynthetic potential [10,17].

Many advantages of LEDs make them an attractive alternative to conventional, fluorescent light. They provide lower heat emission, allow for light intensity and wavelength adjustment, and the lamps have a longer life and occupy a smaller space [10,18]. LEDs emit a more specific light spectrum than standard, fluorescent lamps [10,18]. Their advantage is durability, as well as the ability to precisely match the range of light emitted by the diodes to the plant photoreceptors that may control plant morphogenesis, cellular redox adjustment, or the metabolic pathway of specialized metabolites [10,19,20]. They may enhance plant growth, development, and biomass [10]. In addition, the various light sources (LEDs, fluorescent, or ultraviolet lights) may be used as efficient elicitors to improve the production of biologically active metabolites [18,21].

The present study aimed to investigate, for the first time, the effect of the LED light illumination (blue (BL), red (RL), white (WL), and mixed LED light: red and blue (R:BL) in the ratio 7:3) and cool-white fluorescent lamps (FL) and the cultivation modes (an agar-solidified and liquid-agitated culture) on phenolic compounds accumulation and shoot growth of *R. carthamoides*. It was found that most parameters are significantly influenced by the type of culture and different treatments with LED.

## 2. Results

### 2.1. Effect of the Light Condition on Caffeoylquinic Acid Derivative and Flavonoid Production

**Figure 1:**
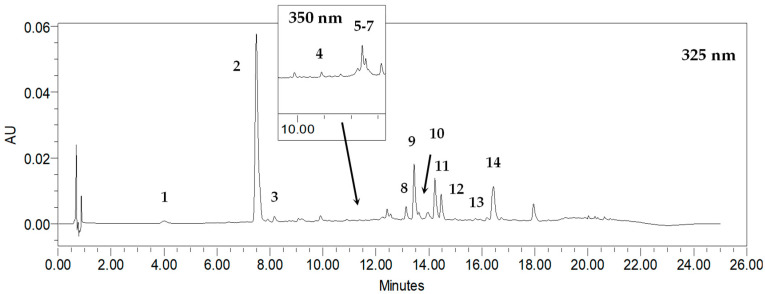
Representative HPLC-UV chromatogram of *R. carthamoides* shoot extract recorded at 325 nm and 350 nm; 3-*O*-caffeoylquinic acid (3-CQA) (**1**); 5-*O*-caffeoylquinic acid (5-CQA, chlorogenic acid) (**2**); 4-*O*-caffeoylquinic acid (4-CQA) (**3**); quercetagetin hexoside (**4**); quercetin hexoside (**5**); luteolin hexoside (**6**); patuletin hexoside (**7**); 3,4-*O*-dicaffeoylquinic acid (3,4-diCQA) (**8**); 3,5-*O*-dicaffeoylquinic acid (3,5-diCQA) (**9**); 1,5-*O*-dicaffeoylquinic acid (1,5-diCQA) (**10**); 4,5-*O*-dicaffeoylquinic acid (4,5-diCQA) (**11**); dicaffeoylquinic acid (di-CQA) (**12**); tricaffeoylquinic acid 1 (tri-CQA 1) (**13**); tricaffeoylquinic acid 2 (tri-CQA 2) (**14**).

**Figure 2:**
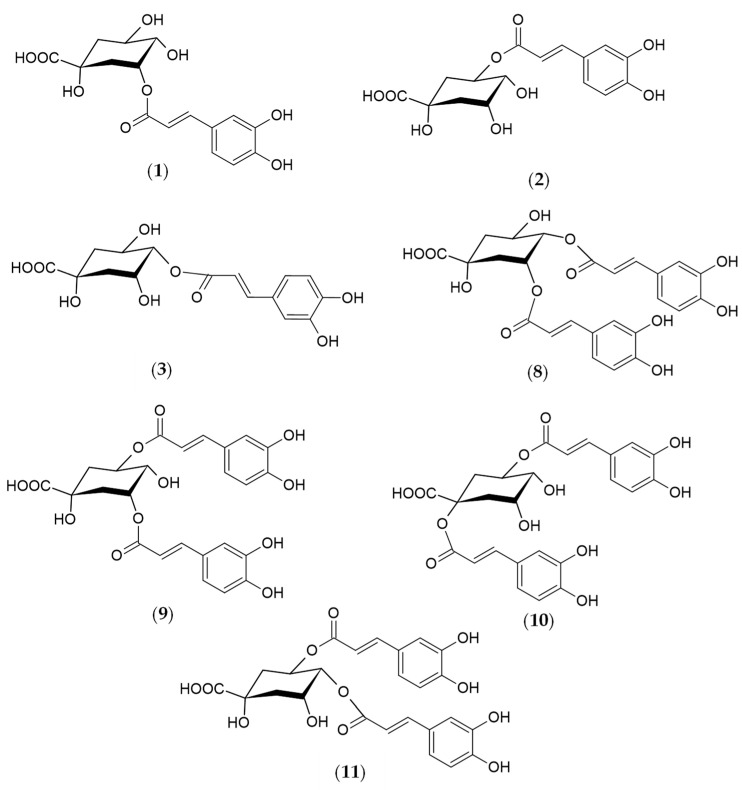
Structures of the phenolic compounds identified in *R. carthamoides* shoots 3-*O*-caffeoylquinic acid (3-CQA) (**1**); 5-*O*-caffeoylquinic acid (5-CQA, chlorogenic acid) (**2**); 4-*O*-caffeoylquinic acid (4-CQA) (**3**); 3,4-*O*-dicaffeoylquinic acid (3,4-diCQA) (**8**); 3,5-*O*-dicaffeoylquinic acid (3,5-diCQA) (**9**); 1,5-*O*-dicaffeoylquinic acid (1,5-diCQA) (**10**); 4,5-*O*-dicaffeoylquinic acid (4,5-diCQA) (**11**).

**Figure 3:**
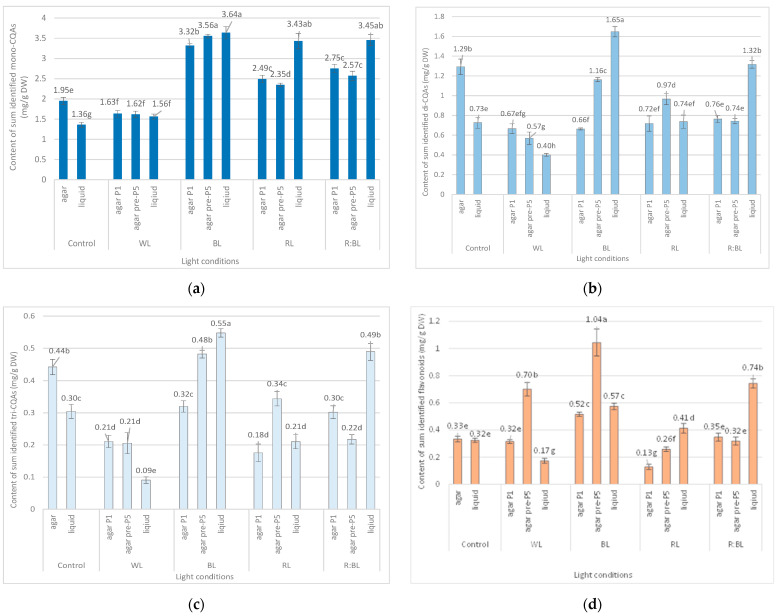
Effect of the LED light conditions on the content of mono-CQAs (**a**), di-CQAs (**b**), tri-CQAs (**c**), and flavonoid monoglycosides (**d**) in *R. carthamoides* shoots cultured for 35 days on an agar (0.7%) and the liquid-agitated MS medium supplemented with IAA 0.1 mg/L and BA 0.5 mg/L. The results are means values ± SE. There is no difference (*p* < 0.05) among the means marked with the same letter. Control—cool-white fluorescent light (FL); WL—white LED light; BL—blue LED light; RL—red LED light; R:BL—red and blue LED (7:3) light; agar—shoots grown on agar (0.7%) MS medium; liquid—shoots grown in liquid MS medium, in a 300 mL Erlenmeyer flask on a rotary shaker at 80 rpm; agar P1—shoots grown on agar (0.7%) MS medium directly exposed to LED lights; agar pre-P5—shoots grown on agar (0.7%) MS medium exposed to LED lights for 5 passages (35 days of each).

**Figure 4:**
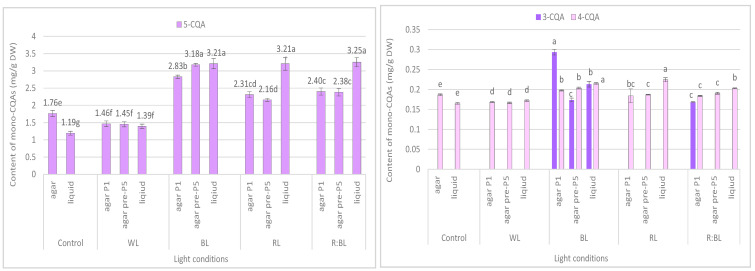
Effect of the LED light conditions on the content of individual mono-CQAs in *R. carthamoides* shoots cultured for 35 days on an agar (0.7%) and in the liquid-agitated MS medium supplemented with IAA 0.1 mg/L and BA 0.5 mg/L. The results are means values ± SE. There is no difference (*p* < 0.05) among the means for the same metabolite marked with the same letter. The compound codes refer to those applied below Figure 1. Abbreviations refer to the conditions of the conducted analysis, as indicated below Figure 3.

**Figure 5:**
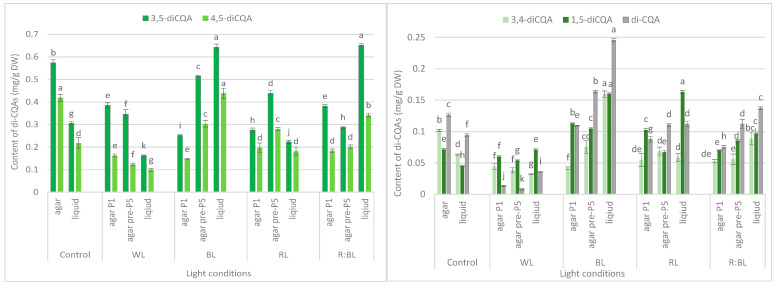
Effect of the LED light conditions on the content of individual di-CQAs in *R. carthamoides* shoots cultured for 35 days on an agar (0.7%) and in the liquid-agitated MS medium supplemented with IAA 0.1 mg/L and BA 0.5 mg/L. The results are means values ± SE. There is no difference (*p* < 0.05) among the means for the same metabolite marked with the same letter. The compound codes refer to those applied below Figure 1. Abbreviations refer to the conditions of the conducted analysis, as indicated below Figure 3.

**Figure 6:**
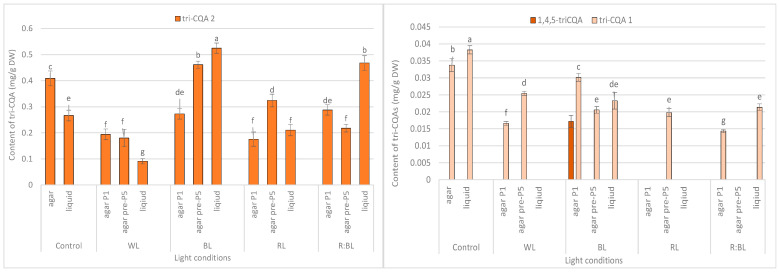
Effect of the LED light conditions on the content of individual tri-CQAs in *R. carthamoides* shoots cultured for 35 days on an agar (0.7%) and in the liquid-agitated MS medium supplemented with IAA 0.1 mg/L and BA 0.5 mg/L. The results are means values ± SE. There is no difference (*p* < 0.05) among the means for the same metabolite marked with the same letter. The compound codes refer to those applied below Figure 1. Abbreviations refer to the conditions of the conducted analysis, as indicated below Figure 3. 1,4,5-triCQA—1,4,5-*O*-tricaffeoylquinic acid.

**Figure 7:**
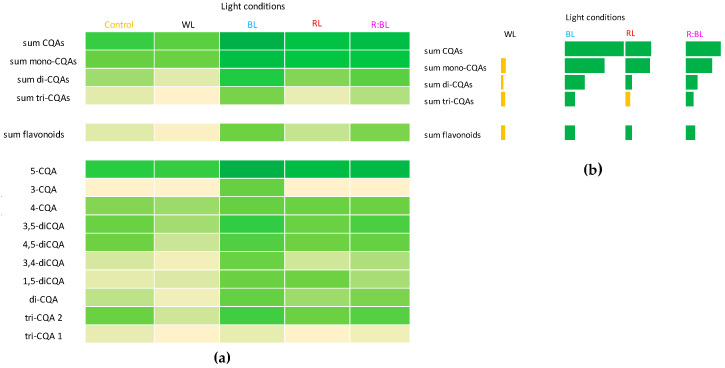
Heat map visualization of CQA and flavonoid productivity (mg/L). The color intensity of boxes indicates the level of specialized metabolites; darker green color presents high content while yellow colors present a low content (**a**). Productivity of CQAs and flavonoids expressed as a level above (green color) or below (yellow color) control (fluorescent light) (**b**). Abbreviations refer to the conditions of the conducted analysis, as indicated below Figure 3. The compound codes refer to those applied below Figure 1.

As a result, eleven phenolic acids were revealed in *R. carthamoides* shoots, according to the previous UPLC-PDA-ESI-MS^3^ study [14]. They were classified as mono-, di-, and tricaffeoylquinic acid derivatives (CQAs). Among the CQAs were identified 3-*O*-caffeoylquinic acid (3-CQA) (**1**), 5-*O*-caffeoylquinic acid (5-CQA, chlorogenic acid) (**2**), 4-*O*-caffeoylquinic acid (4-CQA) (**3**), 3,4-di-*O*-caffeoylquinic acid (3,4-CQA) (**8**), 3,5-di-*O*-caffeoylquinic acid (3,5-diCQA) (**9**), 1,5-di-*O*-caffeoylquinic acid (1,5-CQA) (**10**), 4,5-di-*O*-caffeoylquinic acid (4,5-diCQA) (**11**), and 1,4,5-tri-*O*-caffeoylquinic acid (1,4,5-triCQA) (Figure 1). Figure 2 shows the structures of CQA identified in *R. carthamoides* shoots. Furthermore, dicaffeoylquinic acid derivative (di-CQA) (t_R_ = 14.6 min) (**12**) and two tricaffeoylquinic acid derivatives (tri-CQA 1 (**13**) and tri-CQA 2 (**14**), t_R_ 15.6 min and 16.3 min, respectively) were preliminarily identified. In addition, among other polyphenolic compounds of the shoot extracts flavonoid monoglycosides (derivatives of quercetagetin (**4**), quercetin (**5**), luteolin (**6**), and patuletin (**7**)) were also found. The effect of the time treatment and LED light spectrum was observed on the content of the sum of all detected CQAs and individual specialized metabolites in shoots grown on an agar-solidified and liquid-agitated medium (Figure 3, Figure 4, Figure 5, Figure 6, Figure 7 and Figure S1). The highest total phenolic acid content (sum of all detected CQAs) was found in shoots grown in a liquid system under BL light (5.8 mg/g of dry weight (DW)). The lowest level of the total CQAs was noted in the shoots, which were also cultured in the liquid system but under WL light (2.1 mg/g DW) (Figure S1). As for flavonoids, the shoots cultivated longer under BL light resumed their accumulation. The production of flavonoids was significantly reduced in shoots grown in the liquid medium under WL light (Figure 3d).

#### 2.1.1. Agar Shoot Culture

The content of the sum of all identified mono-CQAs derivatives in an agar shoot culture after 35 days ranged from 1.62 mg/g DW to 3.56 mg/DW and was dependent on the LED light conditions and the treatment time (Figure 3a). Under control conditions, 1.95 mg/g DW of mono-CQAs was found. The accumulation of mono-CQAs was significantly enriched after treatment with BL, followed by R:BL and RL lights. In addition, the mono-CQA content was affected by the time of LED light exposure only to BL and RL lights (Figure 3a). Eventually, the highest level of mono-CQA derivatives was noted under BL light; it was above 1.8-fold higher than that for control shoots. The main mono-CQA was 5-CQA regardless of the light conditions, with the highest amount (3.18 mg/g DW) in the shoot pre-cultured under BL light (Figure 4). Under control conditions, the 5-CQA level was 1.76 mg/g DW. WL light was ineffective for the accumulation of mono-CQAs and 5-CQA. It is worth noting that 3-CQA was detected only in shoots cultured under BL and R:BL lights, the level of which depended on the duration of the light treatment. Of other CQAs, 4-CQA content was similar in shoots grown under BL, RL, R:BL, and the control lights (about 0.2 mg/L) and was slightly higher than that under WL light (0.17 mg/g DW) (Figure 4).

LEDs significantly reduced the sum of all identified di-CQAs and the content of the main di-CQAs, e.g., 3,5-diCQA and 4,5-diCQA, in contrast to the fluorescent lamps (Figure 3b). For example, the level of sum di-CQA in control shoots was 1.29 mg/g DW, but in shoots exposed to LED lights, it ranged from 0.57 mg/g DW to 1.16 mg/g DW. It was observed that the pre-treatment with BL and RL lights enhanced the total content of di-CQA (1.16 mg/g DW and 0.97 mg/g DW), but the level was lower than that under control conditions (1.29 mg/g DW) (Figure 3b). A similar trend was observed for 3,5-, 4,5-, and 3,4-diCQA. In contrast, BL light was most favorable for the accumulation of 1,5-diCQA or tentatively identified di-CQA, whereas WL light significantly reduced the concentration of this di-CQA (Figure 5).

Tri-CQAs were enhanced in the shoots pre-cultured under BL light (0.48 mg/g DW vs. 0.32 mg/g DW) and RL light (0.34 mg/g DW vs. 0.18 mg/g DW). However, the content of tri-CQAs noted in shoots exposed to RL light was lower than that recorded under control, FL light (0.44 mg/g DW) (Figure 3c). The main tri-CQA was tri-CQA 2, which accounted for over 95% of all tri-CQAs. In addition, 1,4,5-triCQA was found only in shoots maintained for one passage under BL light (0.02 mg/g DW) (Figure 6).

The level of flavonoids in response to LED light treatments was higher in shoots pre-treated with BL light (1.04 mg/g DW); it was two- and three-fold more significant than that for shoots directly exposed to BL and FL lights, respectively (Figure 3d). The increase in the flavonoid content in shoots pre-treated with LED lights was also noted under WL and RL lights, but only WL light enhanced the content of flavonoids (0.7 mg/g DW) compared to control lamps (0.33 mg/g DW). The RL light significantly reduced flavonoid accumulation regardless of exposure time (to 0.13 and 0.26 mg/g DW) (Figure 3d).

#### 2.1.2. Liquid-Agitated Shoot Culture

The liquid-agitated shoots accumulated the same CQA derivatives as the agar shoot culture except for 1,4,5-triCQA. The total level of all identified mono-CQAs and 5-CQA under R:BL and RL lights increased about 1.5 times in the liquid-agitated shoot culture in contrast to agar-solidified shoots grown under the same light conditions and reached the content of about 3.5 mg/g DW (Figure 3a and Figure 4). However, these contents were similar to that under BL lights and achieved the maximal value—3.64 mg/g DW for total mono-CQAs and 3.21 mg/g DW for chlorogenic acid. The WL light did not affect the level of these phenolic acids in liquid shoots compared to agar shoots. In the control shoots, the levels of mono-CQA and 5-CQA were observed to decrease. The liquid medium also stimulated the production of 4-CQA, primarily when shoots were grown under BL, RL, and R:BL lights. The highest content of this phenolic acid was noted under RL and BL lights (0.22 mg/g DW) (Figure 4). Notably, 3-CQA was detected only in shoots cultured under BL light (0.21 mg/g DW).

The BL and R:BL lights and the liquid medium also stimulated the production of di-CQAs (Figure 3b). The maximal content of these phenolic acids was achieved under BL light (1.65 mg/g DW), followed by R:BL light (1.32 mg/g DW); it was about two-fold higher than in the control shoots. The main di-CQA was 3,5-CQA, whose content was maximized by the treatment with BL and R:BL lights (0.65 mg/g DW). The peak level of 4,5-diCQA was found under BL light (0.44 mg/g DW), followed by R:BL light (0.34 mg/g DW) (Figure 5). Additionally, BL and R:BL lights stimulated the accumulation of 3,4-, 1,5- and tentatively identified di-CQA, while RL enhanced the content of 1,5-diCQA.

Similarly, the content of tri-CQAs increased under BL and R:BL lights (Figure 3c). For example, the total content of tri-CQAs in agitated shoots under R:BL light was about two-fold higher than in agar shoots (0.49 mg/g DW vs. 0.22 mg/g DW and 0.30 mg/g DW). The highest level of tri-CQAs was recorded under BL light (0.55 mg/g DW). The tentatively identified tri-CQA 2 dominated in the plant extracts regardless of the light conditions with the highest content under BL and R:BL lights (0.52 mg/g DW and 0.47 mg/g DW, respectively) (Figure 6). In contrast, fluorescent lamps, RL, and WL lights significantly lowered the content of tri-CQA 2 to 0.27 mg/g DW, 0.21 mg/g DW, and 0.09 mg/g DW, respectively.

As for the flavonoids, the accumulation of these specialized metabolites was enhanced under R:BL, followed by BL, and RL lights with the maximal level under R:BL light (0.74 mg/g DW); it was two-fold higher than that for agar shoot culture grown in the same conditions and control shoots (about 0.35 mg/g DW) (Figure 3d).

The CQA and flavonoid productivity expressed in mg/L in *R. carthamoides* liquid-agitated shoots under different LED light wavelengths was also characterized (Figure S2) and presented graphically in a heat map (Figure 7a). In addition, Figure 7b illustrates the productivity of specialized metabolites above (green color) and below (yellow color) control. The yield of CQAs and flavonoids was strongly enhanced by exposure to BL light, followed by R:BL and RL lights, while WL light decreased the yield of phenolic compounds (Figure 7 and Figure S2). The highest CQA (68 mg/L) and flavonoid yields (6.7 mg/L) were found under BL light when the shoots achieved the highest DW. The greatest productivity of the most abundant CQAs, chlorogenic acid (37 mg/L), was also reported under BL light (Figure 7).

### 2.2. Effect of LED Light Condition on R. carthamoides Shoot Growth and Biomass Increment

#### 2.2.1. Agar-Solidified Shoot Culture

**Figure 8:**
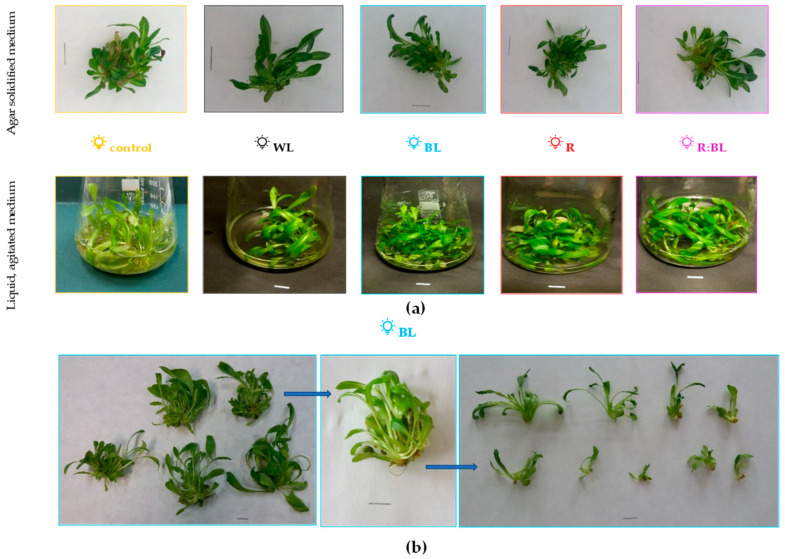
*R. carthamoides* shoots cultured for 35 days on an agar (0.7%) solidified and in the liquid, agitated MS medium under various light conditions: control—cool-white fluorescent light (WFL), WL—white LED light, BL—blue LED light, RL—red LED light, R:BL—red and blue (70%:30%) LED light (**a**). The shoots multiplied in the liquid-agitated MS medium under BL light for 35 days (**b**). The media were supplemented with IAA 0.1 mg/L and BA 0.5 mg/L. Bar = 1 cm.

**Figure 9:**
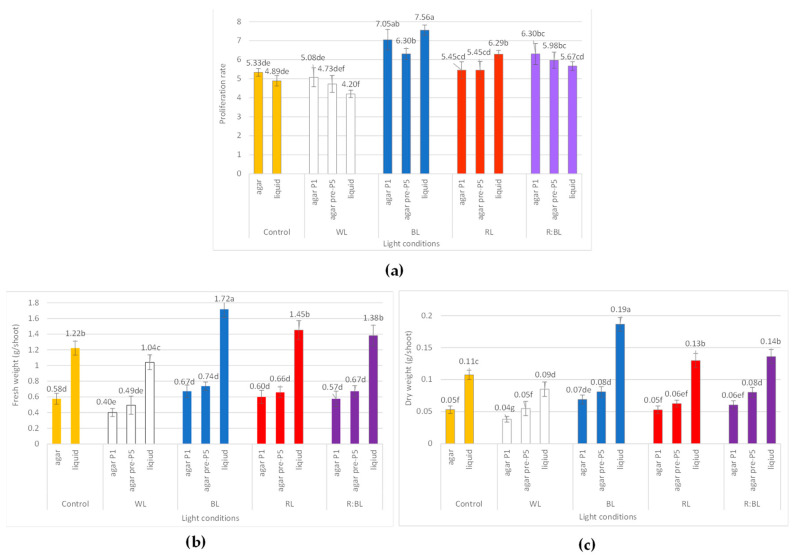
The effect of LED light conditions on the growth of *R. carthamoides* shoots: (**a**) proliferation rate; (**b**) fresh weight (FW) (g/shoot); (**c**) dry weight (DW) (g/shoot). The results are mean values ± SE. There is no difference (*p* < 0.05) among the means marked with the same letter. Abbreviations refer to the conditions of the conducted analysis, as indicated below Figure 3.

Although exposure to LED lights influenced the number of new shoots and buds, the duration of the light treatment did not significantly affect the number and the length of *R. carthamoides* shoots (Figure 8a, Figure 9a, and Figure S3).

The shoot micropropagation rate ranged from 4.73 to 7.05, and the best proliferation rate was achieved under BL light (Figure 9a). The exposure to mixed R:BL light also had a stimulating effect on the shoot multiplication. LED lights, mainly BL, RL, and R:BL lights, positively affected the number of multiplied structures; cultures developed more shoots compared to the buds (66.8–67.89%). In addition, more prolonged exposure of shoots to BL, RL, and R:BL lights decreased the buds-to-shoots ratio (Figure S3a).

The light exposure time and LED light spectrum influenced shoot morphology—the shoot quality and height (Figure S3). The shoots pre-treated with LED lights showed better morphology except for RL light; the cultivation for a longer period lowered hyperhydricity frequency (15.56–35.89% vs. 18.58–42.83%) (Figure S3b). In addition, the shoots cultured under BL, RL, and R:BL lights demonstrated better general conditions than those under WL; more shoots had correct morphology and were longer.

On the other hand, LED lights harmed the shoot length (Figure S3c). The shoots under control conditions reached almost 2.5 cm in length, while those under LEDs were about two-fold shorter (1.2–1.6 cm).

Although LED light exposure (BL, RL, and R:BL lights) improved the proliferation rate, there was no significant difference in the fresh weight (FW) of the shoot compared to the control condition (Figure 9b). Compared to shoots grown without prior adaptation to light, the more extended shoot cultivation under LED lights was also not statistically significant in terms of the dry weight except for R:BL and WL lights (Figure 9c). DW was affected by BL, R:BL, and WL lights compared to FL light. The best result was observed under BL and R:BL lights, reaching 0.07–0.08 g/shoot of DW.

#### 2.2.2. Liquid-Agitated Shoot Culture

*R. carthamoides* shoots cultured in a liquid, stationary medium turned up and died after two weeks. Therefore, the shoots were multiplied in an agitated medium. The proliferation rate ranged between 4.20 and 7.56, depending on the light conditions, and reached the highest value under BL light (Figure 9a). The liquid-agitated medium also had a beneficial effect on the morphology of shoots regardless of the light irradiations (Figure 8 and Figure S3). Only 9.41% showed hyperhydricity symptoms under BL light, which was two-fold less than on agar medium under the same light condition (Figure S3b). In addition, the shoots were much longer than those from solid media, independently of LED lights. The most stimulated effect on the shoot length was found under BL light. The shoots reached 3.4 cm long (vs. 1.6 cm on an agar medium) (Figure 8b and Figure S3c).

It was found that the fresh weight of *R. carthamoides* shoots cultured in the liquid medium was improved in comparison to an agar-solidified medium and ranged from 1.04 g/shoot to 1.72 g/shoot. In addition, exposure to LED lights (except for WL light) stimulated FW; the maximal FW was noted under BL light (Figure 9b). The dry weight of multiplied shoots was also enhanced in a liquid medium regardless of the light conditions (Figure 9c). The best result was observed under BL light (0.19 g/shoot), followed by R:BL and RL lights (0.13 and 0.14 g/shoot, respectively), FL light (0.11 g/shoot), and WL light (0.09 g/shoot).

## 3. Discussion

The plant receives light signals through photoreceptors that absorb a broad spectrum of light with a specific wavelength of <400 nm (UV radiation), 400–700 nm (visible), and 700–800 nm (far-red) [22]. Light is necessary for photosynthesis and is associated with the developmental response, morphogenesis, and phototropism in plants [23]. Numerous photoreceptors, e.g., cryptochromes, phytochromes, and phototropins, regulate plant morphogenesis [24]. Phytochrome absorbs red and far-red light spectra. Cryptochrome is a blue light photoreceptor [25].

White, fluorescent lamps (with 350–750 nm wavelengths and spectral emission) are often used in in vitro cultures. Their disadvantages are their large size and relatively high energy consumption [10], which generates high production costs [22]. In addition, they induce high radiant heat. A more economical option is to use LEDs, characterized by a wider variability of spectral wavelength than fluorescent lamps and less energy consumption [10,26]. LEDs are smaller in size and weight and have a long life; therefore, they are cheaper to use, more durable, do not contain mercury, and do not emit thermal energy or show low heat emission [10,18,24]. The type of light (light source), light intensity, wavelength, exposure duration, or photoperiod [23,27] may influence plant growth, development, or metabolic pathway. Light wavelength can control plant growth by regulating endogenous growth regulators. Phytochrome-interacting factors (PIFs) and long hypocotyl 5 (HY5) mediate the interaction between light and hormone-signaling pathways such as auxin, cytokinin, abscisic acid, gibberellin, and ethylene [28,29]. However, too much or too little light, as well as the day–night photoperiod can stress the plant. High light levels can cause photoinhibition of photosynthesis [30].

To the best of our knowledge, there are no reports on the effect of LED light quality and cultivation modes on the growth and specialized metabolite production by *R. carthamoides* shoots micropropagated in vitro. The present study demonstrated that the type of culture, LEDs, and duration of light treatment may affect both the CQA and flavonoid contents and the growth of shoots. Our findings demonstrated that the liquid medium and blue LED light were most beneficial for the growth of *R. carthamoides* shoots, propagation, biomass, and CQAs accumulation.

In plant in vitro cultures, optimizing the culture conditions regarding biomass and the biosynthesis of specialized metabolites is essential to obtain high productivity of individual, active metabolites. The light quality can change endogenous phytohormone levels and may regulate the secondary metabolism in the plants [31]. The light irradiance, wavelength, and duration of light exposure as the stressor factors [27] may also alter the physiology of plant cells in in vitro cultures; therefore, it may affect the biosynthesis pathway and enhance the production of the specialized metabolites in plant cultures. Thus, the effect of LED illumination spectra on the biosynthesis of specialized metabolites in *R. carthamoides* shoot cultures was also performed. Previous studies confirm that CQAs are one of the primary specialized metabolites detected in *R. carthamoides* [1], which exhibit a broad spectrum of biological activity [32]. In addition, the plant material obtained in vitro was also a rich source of these phenolic compounds [13,14,15,16].

In the present study, it was found that the liquid-agitated medium improved the dry weight of *R. carthamoides* shoots regardless of the light conditions. For example, after 35 days, DW increment was two-fold higher in the liquid medium (0.11 g/shoot) than that on an agar-solidified medium (0.05 g/shoot) under control, fluorescent lamps and may be associated with the fact that the shoots maintained in a liquid medium were longer and formed a higher number of new shoots than buds compared with those in the agar. This phenomenon may be due to the easier distribution of dissolved components or oxygen. Several studies have reported that the liquid medium, stationary or agitated, also promoted biomass of in vitro cultured shoots of *Boswellia serrata*, *Celastrus paniculatus* [33], *Schisandra chinensis* [34], and *Spiraea betulifolia* ssp. *aemiliana* [35]. The LED lights also promoted the biomass accumulation of *R. carthamoides* shoots cultured in a liquid-agitated medium. The highest increase in DW (0.19 g/shoot) was achieved under BL light. Exposure to BL light appears to be more effective in DW increment of other plant species such as *Achillea millefolium* [36], *Scrophularia kakudensis* [37], and *Stevia rebaudiana* [38]. The study of Kyriacou et al. [39] also confirmed that the influence of the light wavelength on biomass is species-specific. For example, BL or RL light (supplemented with 10% green-yellow light) enhanced the DW of *Lepidium sativum* cv. Curled microgreens, BL:RL light (45% RL, 10% green light, and 45% BL) was most favorable for the dry increment of *Amaranthus tricolor* cv. Red garnet microgreens, whereas different light spectra did not affect biomass of *Brassica rapa* var. *japonica* cv. Greens and *Portulaca oleracea* microgreens.

The total contents of all identified CQAs, flavonoids, and individual specialized metabolites in *R. carthamoides* shoots were affected by the type of medium, the LED light conditions, and the time of light exposure. Generally, the liquid medium was more favorable to phenolic compound production than an agar-solidified medium. The content of sum CQAs after 35 days in shoots cultured in an agar-free medium under different light conditions ranged from 2.1 mg/g DW to 5.8 mg/g DW. The highest increase (2.5-fold) was found in the presence of BL light. After one month, all identified phenolic acids and flavonoids yielded 68 mg and 7 mg per liter of medium, respectively. In comparison, the content of total CQAs in the leaves of seed-derived plants after 3-month growth in pots in the greenhouse was above two-fold lower [15].

The liquid, agitated shoots accumulated the same CQA derivatives as the agar shoot culture except for 1,4,5-triCQA. Following our previous studies [14,15,16], *R. carthamoides* shoots cultured on an agar-solidified and in liquid-agitated media also dominated mono-CQAs with 5-CQA as the main component regardless of the type of medium and LED light illuminations. The liquid-agitated medium was more favorable for 5-CQA accumulation than agar. It was similar to the research of Zheleznichenko et al. [35]. In *Spiraea betulifolia* ssp. *aemiliana* microshoots, the content of 5-CQA was found to be 1.5-fold lower in an agar-solidified than a liquid system [35], while the level of 5-CQA in microshoots of *Schisandra chinensis* cv. Sadova No. 1 [40] and *Eryngium alpinum* [41] was enhanced in the solid medium.

The mono-CQA level in *R. carthamoides* shoots was significantly enhanced under BL and RL lights and the combination of RL and BL lights (7:3) compared to the control condition. The production of 5-CQA ranged from 1.2 mg/g DW to 3.2 mg/g DW and was strongly enhanced by LED lights or the liquid medium. The highest concentration of this mono-CQA was noted in shoots grown in a liquid medium treated with R:BL, BL, and RL lights (about 3 mg/g DW). The 5-CQA content was similar to the level found in *R. carthamoides* pRi-transformed plants (3.3 mg/g DW) [15] and 3.4-fold higher than that in plants regenerated in vitro by indirect organogenesis (0.94 mg/g DW) [13] growing in a greenhouse for three months. BL light was also the most favorable LED light for 5-CAQ accumulation in *Lactuca sativa* cv. Green Wave plants grown under continuous lighting [42]. A promising result after treatment with BL or RL light for an increment of 5-CQA level in *R. carthamoides* shoots in the present study was also confirmed in *Amaranthus tricolor* cv. Red garnet, *Lepidium sativum* cv. Curled, *Portulaca oleracea* microgreens [39], and strawberry seedlings [43]. A significant increase of 5-CQA after RL:BL light (70%:30%) postharvest treatment was also noted in *Solanum melongena* fresh fruits [44]. In contrast, Morańska et al. [45] did not observe the effect of different LED light spectra on 5-CQA accumulation in *Leucojum aestivum*. It should be stressed that another mono-CQA, 3-CQA, was accumulated only in *R. carthamoides* shoots treatment with BL light (regardless of the type of culture) with the highest content in shoots directly exposed to LED light.

LED irradiations significantly reduced the sum di-CQAs and the content of the main di-CQAs, e.g., 3,5-diCQA and 4,5-diCQA in *R. carthamoides* shoots cultured on an agar-solidified medium. In contrast, BL and R:BL lights positively affected di-CQAs accumulation in shoots grown in the liquid-agitated medium. The highest content of di-CQAs (1.65 mg/g DW) was found under BL light. The agitated shoots growing under R:BL light also produced a high content of di-CQAs (1.32 mg/g DW). Among di-CQAs, 3,5- and 4,5-CQA dominated, and their levels were highest in agitated shoots grown under BL (0.64 mg/g DW and 0.44 mg/g DW, respectively) and R:BL lights (0.65 mg/g DW and 0.34 mg/g DW, respectively). In the present study, in *R. carthamoides* shoots (regardless of lighting conditions), 1,3-diCQA was absent while it was accumulated in seed-derived plants and pRi-transformed plants of *R. carthamoides* [15]. In addition, the transformed roots also produced 1,3-diCQA [14,16], whose content depended on the light conditions (dark or fluorescent cool white lamps). A higher level was detected in roots cultured in the dark [14], and maybe the light is not beneficial for accumulating this phenolic acid in this organ.

The tri-CQAs level in *R. carthamoides* shoots was also significantly different among the tested LED lights with BL as the most effective condition, especially for shoots cultured in the liquid medium (0.55 mg/g DW). The tentatively identified tri-CQA 2 derivative was the main compound among tri-CQA derivatives (0.09–0.52 mg/g DW), which also dominated in leaves of transformed plants and seed-derived plants but with a higher concentration [15]. Notably, 1,4,5-triCQA was identified only in agar-solidified shoots directly exposed to BL light (0.02 mg/g DW). This compound was also found in the leaves of *R. carthamoides* transformed plants and transformed roots in previous studies [14,15,16].

The duration of LED light exposure affected mono-CQAs accumulation in *R. carthamoides* shoots. The content of mono-CQAs was higher after a more prolonged exposure to BL. Furthermore, the pre-treatment with BL and RL lights also increased di-CQA content (from 0.66–0.72 mg/g DW to 0.97–1.16 mg/g DW), which was, however, not higher than for control conditions (1.29 mg/g DW). The pre-treatment with BL and RL lights also enhanced tri-CQAs level (from 0.18–0.32 mg/g DW to 0.34–0.48 mg/g DW). Similarly, Park et al. [46] observed that the content of some specialized metabolites (rosmarinic acid and tilianin) in *Agastache rugosa* seedlings varied on the exposure time of LED lights. The rosmarinic acid level increased with time, and the highest amount was noted at three weeks under WL light. In the present study, it was not found that the duration of treatment with different LED lights enhanced the content of 5-CQA in *R. carthamoides* shoots. Similarly, the level of chlorogenic acid in *Lactuca sativa* var. Lollo Rossa and *Ocimum basilicum* cv. Genoverse Gigante was not enhanced in plants when the different periods of BL light (0–48 days) were used [47]. On the other hand, the accumulation of 5-CQA in *Lactuca sativa* cv. Green Wave plants was influenced by different photoperiodic conditions (12/12, 14/10, 16/8, 20/4, 24/0h light/dark) and increased with BL light duration. The maximal value (9.6 mg/g DW) was noted after 24/0 treatment. In addition, the highest content of 5-CQA was also observed at 54 h after continuous BL treatment (over 13 mg/g DW), and it was over two-fold higher than that under steady RL light [42]. The accumulation of 5-CQA in sprouts of *Fagopyrum tataricum* cv. Hokkai T8’ decreased during the 10-day time exposure to light, and the highest value was found two days after exposure to BL and WL lights [48].

The positive effect of the liquid system for flavonoid production was noted for *R. carthamoides* shoots exposed to R:BL light, followed by BL and RL lights. However, only R:BL and RL lights elicited the content of these compounds compared to the shoots cultured on agar medium in the same light conditions, but the highest level of flavonoids demonstrated *R. carthamoides* shoots pre-cultured on a solid medium under BL light (1.04 mg/g DW). The agar media also favored flavonoid production in *Scutellaria lateriflora* microshoots [49]. According to our results, BL light led to the up-regulation of flavonoid biosynthesis in other plant species, for example, *Ajuga bracteosa* adventitious roots [50], *Curculigo orchioides* synseed-derived seedlings [51] and *Scrophularia kakudensis* shoots [37]. Exposure to BL light of *Operculina turpethum* [52] and *Rhodiola imbricata* [53] callus culture also positively affected specialized metabolite production. It resulted in a higher content of flavonoids and total phenolics than other LED light illuminations.

The intensity of LED light irradiation can also affect the accumulation of specialized metabolites. In the present study, LED and fluorescent light intensity was about 40 µmol m^−2^ s^−1^. The content of total phenolic compounds and total flavonoids in *Urtica dioica* plantlets in vitro was improved with an increase in the intensity of white LED light, from 26 µmol m^−2^ s^−1^ to 130 µmol m^−2^ s^−1^ [54]. Similarly, the accumulation of cardenolide in *Digitalis mariana* ssp. *heywoodi* plants enhanced with increased white LED light intensity (20–139 µmol m^−2^ s^−1^) [55] while the high intensity of BL light, 200 µmol m^−2^ s^−1^, down-regulated nineteen genes involved in the flavonoid pathway in young tea plant *Camellia sinensis* cv. Fujian Shuixian [56].

Combining blue and red light can stimulate specialized metabolite accumulation, especially when the ratio of red to blue LED light is 3:1 or higher [57,58]. The total phenolic content in *Valerianella locusta* plants cultivated in the greenhouse was significantly enhanced by the high R:BL ratio (9:1) [57]. In contrast, the ratio 1:2.5 of RL to BL LED light led to an increase in the total phenolic compound and flavonoid level in *Urtica dioica* plantlets [54], while an equal ratio of red and blue LED illumination improved the accumulation of 20-hydroxyecdysone in *Pfaffia glomerata* shoots [59]. The higher ratio of red light (3RL:1BL) and blue light (1RL:3BL) resulted in the lowest accumulation of this ecdysteroid [59].

The elevated CQA and flavonoid levels under BL light in *R. carthamoides* shoots are most likely because of the higher energy levels of the short wavelength of blue light. Thus, it possibly stimulates oxidative stress that, as the stress-generating factor, elicits the accumulation of specialized metabolites in plants. Furthermore, the changes in the content of the phenolic compounds in shoots treated with different LED lights may be associated with the effect of the gene expression elaborated in the secondary metabolism. Some literature reported that LED lights, especially blue light, can enhance the expression of the primary genes involved in the phenylpropanoid pathway, such as phenylalanine ammonia-lyase (PAL), hydroxycinnamoyl-CoA shikimate hydroxycinnamoyltransferase (HCT), 4-coumaryl-CoA ligase (4CL), flavonol synthase (FLS), dihydroflavonol-4 reductase (DFR), chalcone isomerase (CHI), chalcone synthase (CHS), and flavanone 3-hydroxylase (F3H) [17,43,46,48,60]. However, the mechanism by which LED light treatment stimulates the content of CQAs and flavonoids in *R. carthamoides* shoots is unclear, and further research needs to be conducted.

In conclusion, the CQA and flavonoid yields were up-regulated under BL light, followed by R:BL and RL lights, while WL light decreased the yield of phenolic compounds in *R. carthamoides* shoots. After 35 days of culture under BL light, 68 mg of CQAs and 7 mg of flavonoids from one liter of medium can be obtained. The greatest productivity of the most abundant CQAs, chlorogenic acid, was also reported under BL light.

An agar-free medium was used to reduce the cost of *R. carthamoides* shoot growth caused by the high price of agar. It was found that the liquid system enhanced the accumulation of phenolic compounds in *R. carthamoides* shoots, affected faster culture growth and stimulated biomass increment or specialized metabolite production. Compared to the agar culture, using a liquid medium allows for the application of much larger cultivating containers. Furthermore, the scale of the shoot culture can be increased by bioreactor cultivation, which may automate the propagation process.

The present study demonstrated that the medium type did not affect the proliferation rate of *R. carthamoides* shoots except for RL light. Both tested media were beneficial to shoot micropropagation. A significant promotion in the number of *R. carthamoides* shoots and buds was found when shoots were exposed to LED lights and BL light was the most favourable for the shoot propagation The liquid medium stimulated shoot growth of *Boswellia serrata* [33], *Catharanthus roseus* [61], *Celastrus paniculatus* [33], and *Centaurium erythraea* [62] while BL or mixed red and blue (ratio 1:1) lights enhanced the number of new shoots of *Achillea millefolium* [36] and *Ficus benjamina* “Exotica” shoots [63]. The duration of the light exposure did not affect the proliferation rate of *R. carthamoides* shoots, but it was found that the shoots’ pre-treatment with LED lights showed better morphology; culturing for extended periods lowered the frequency of hyperhydricity shoots and the proportion of buds to shoots.

The advantage of using an agar-free medium may be cost reduction and the fact that *R. carthamoides* shoots exhibited generally better conditions. The frequency of hyperhydricity structures was lower in this type of culture. Numerous reports demonstrated adverse effects on agar for micropropagation of many plant species for medicinal purposes [33,61,62]. An agar may chelate or adsorb nutrient ions; therefore, they are weaker or unavailable to the explants [61]. The gelling agents may inhibit the growth of shoots or may generate hyperhydricity syndrome [64]. Hyperhydricity is not a desirable phenomenon in plant in vitro culture because it lowers the quality of the material (shoot demonstrates morphological abnormalities) [64,65]. The positive effect on *R. carthamoides* shoot quality was observed after treatment with LED lights, mainly BL, RL, and R:BL lights. In addition, pre-treatment with LED lights (except for RL light) also lowered the hyperhydricity symptoms of shoots. The shoots mentioned in liquid medium under BL light showed the best quality. A few studies also confirm that light conditions can reduce the hyperhydricity problem [64,66,67].

Although the LED light reduced the hyperhydricity symptoms of the *R. carthamoides* shoot, it harmed the length of the shoot multiplied on an agar-solidified medium. The shoots under control conditions reached almost 2.5 cm in length, while those under LED lights were shortened to 1–1.5 cm. The shoot elongation was strongly promoted by the liquid medium. The shoot length of *Catharanthus roseus* [61] and *Eryngium alpinum* [41] was also enhanced when the stationary or agitated liquid medium was used. In addition, the elongation of *R. carthamoides* shoots cultured in the liquid medium was also stimulated by BL light. Enhancement of stem elongation by BL light has also been reported in several plant species, e.g., *Achillea millefolium* [36], *Brassica eruca* “Rocket” [68], and *Vanilla planifolia* [69,70]. The elongation of *R. carthamoides* shoots may be due to the regulation of biosynthesis of endogenous phytohormones such as indole-3-acetic acid (IAA) or gibberellic acid by LED light regimes. Exposure to the blue light of *Platycodon grandiflorum* plantlets [71] and Norway Spruce seedlings [31] increased the level of IAA, and the content of this auxin was significantly higher than that treated with RL light. As for *R. carthamoides* cultures, further research is needed to explore this mechanism.

In conclusion, the liquid medium and blue LED light are the best systems for *R. carthamoides* shoot propagation and may be an alternative method to the conventional cultivation of this rare species.

## 4. Materials and Methods

### 4.1. Plant Materials

*R. carthamoides* shoot culture was obtained as described by Skała et al. [13]. The material for the initiation of *R. carthamoides* shoot culture were seeds obtained from the Medicinal Plant Garden of the Department of Pharmacognosy, Medical University of Lodz (Poland). The voucher specimen (S_RC_014) was deposited at the Department of Biology and Pharmaceutical Botany, Medical University of Lodz (Poland). In the present study as the explant was used the fragment of the stem with axillary bud obtained from shoots cultured on Murashige and Skoog (MS) [72] agar (0.7%) (Duchefa Biochemie B.V., Haarlem, The Netherlands) medium supplemented with 6-benzyladenine (BA) (0.5 mg/L) and indole-3-acetic acid (IAA) (0.1 mg/L) (Duchefa Biochemie B.V., Haarlem, The Netherlands) under a 16h photoperiod, Photosynthetic Photon Flux Density (PPFD) of 40 µmol m^−2^ s^−1^ irradiated by fluorescent lamps at 26 ± 2 °C and subcultured at a 35-day interval.

### 4.2. Effect of the Cultivation Mode (Agar and Liquid Media) and the Light Condition on the Shoot Growth

**Figure 10:**
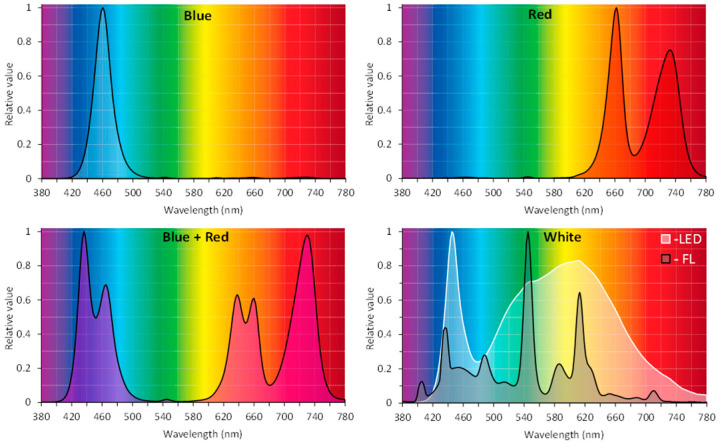
Relative spectral characteristics of light emitted by blue (BL) LED, red (RL) LED, blue + red (R:BL) (70%:30%) LED, white (WL) LED, and control fluorescent lamps (FL).

*R. carthamoides* shoots were multiplied on solidified agar (0.7%) or liquid MS medium supplemented with BA (0.5 mg/L) and IAA (0.1 mg/L). The shoots were grown in a glass tube—180 × 25 mm containing 25 mL of medium (an agar shoot culture) and in a 300 mL flask containing 80 mL of medium on a rotary shaker at 80 rpm (the liquid shoot culture). The optimal type and concentration of phytohormones on the shoot proliferation were selected based on separate experiments. The shoot cultures were grown for five weeks in a growth chamber at 26 ± 2 °C, under four LED light quality combinations: blue (BL) (460 nm), red (660 nm and 730 nm) (RL), a mix of red and blue (70%:30%) (R:BL), and white light (3900K, neutral white) (WL) (PXM Sp., Niepołomice, Poland), at PPFD of 35 µmol m^−2^ s^−1^ and 16/8-h light/dark photoperiod. Light parameters were measured using a spectroradiometer GL Spectis 1.0 Touch + Flicker (GL Optic). Parallelly, the shoots were cultured under a cool-white, fluorescent lamp (control; FL) (OSRAM SMART LUX PRO L 36W/865 Cool Daylight); PPFD of 40 μmol m^−2^ s^−1^ (16/8-h light/dark photoperiod). Relative spectral characteristics of light emitted by BL LED, RL LED, R:BL LED (70%:30%), WL LED, and control fluorescent lamps (FL) are shown in Figure 10. The agar shoot culture was exposed to different light conditions without their prior adaptation to different LED regimes (agar P1). The shoots were also previously acclimatized to different lighting conditions for five 35-day passages (agar pre-P5).

After 35 days of culture, the following parameters were determined: the multiplication rate (the mean number of shoots and buds per explant) and biomass accumulation (fresh weight (FW) g/shoot and dry weight (DW) g/shoot). In addition, the length of shoots (determined as the length of the longest leaf), the percentage of hyperhydricity shoots, the percentage of buds (structures ≤ 0.5 cm), and the percentage of shoots up to 1 cm, 2 cm, 3.5 cm, 5.5 cm long, and above 5.5 cm were also reached (Supplementary Materials, Figure S3). The DW was estimated after lyophilization using freeze dryer Alpha 1–2 LDPlus (M. Christ Gefriertrocknungsanlagen GmbH, Osterode am Harz Germany). The experiment was repeated three times, and 10–15 shoots were used for each treatment; for agar cultures—one explant per tube; for liquid cultures—five explants per flask.

### 4.3. Influence of the Light Condition on Caffeoylquinic Acid Derivatives and Flavonoids

The contents of CQAs and flavonoid glycosides were determined in 80% (*v*/*v*) aqueous methanol extracts of shoots cultured under four LEDs and fluorescent conditions for five weeks. The plant material extraction and the qualitative and quantitative analysis were conducted as described earlier by Skała et al. [14]. Compounds were identified using UPLC-PDA-ESI-MS^3^ and by comparison of their UV-Vis spectra and the retention times according to our earlier study [14]. The quantification of chlorogenic acid isomers, dicaffeoylquinic acid isomers, tricaffeoylquinic acid and its derivative, and flavonoid monoglycosides were based on the calibration curve of chlorogenic acid, cynarin, and isoquercitin, respectively. The contents of all compounds were expressed in mg/g DW. The results are means from nine replicates. In addition, the productivity of the specialized metabolites was expressed in mg/L of medium (for shoots cultured in the liquid medium). The productivity of CQAs and flavonoids was also presented graphically in the heat map (Figure 7a). In addition, Figure 7b shows the productivity of specialized metabolite above (green color) and below (yellow color) control.

### 4.4. Statistical Analysis

The results are calculated as means ± SE. Firstly, the normality of the data was determined by the Shapiro–Wilk test. The results of the treatments were compared by using one-way analysis of variance (ANOVA) with Tukey’s post hoc test (*p* < 0.05) (STATISTICA 13.3PL StatSoft, Krakow, Poland). The heat map visualization and visual effect of comparing values with the control were created based on metabolite content (mg/L of medium) using Microsoft Office Excel.

## 5. Conclusions

The results of the present study demonstrated that light is a stress factor that alters secondary metabolic pathways and shoot growth of *R. carthamoides* shoots. This is the first report documenting the biosynthetic potential of *R. carthamoides* shoots cultured in vitro. Our findings presented that using a liquid system and blue LED is also an efficient technique for accumulating health-promoting CQA derivatives. Liquid systems are also most efficient in obtaining plant materials. In addition, LED illumination spectra, primarily blue light, were the most profitable approaches for the growth of *R. carthamoides* shoots from the stem fragment with axillary bud. The advantages of using a liquid medium are promising to increase the scale of the culture of this rare and endangered species in bioreactors in commercial production. Using a liquid medium will reduce cultivation costs, while LEDs are more economical than fluorescent lamps because they consume less energy, have a longer lifetime, and have low thermal energy emissions, which makes them more environmentally friendly.

## Data Availability

Data are contained within the article.

## Supplementary Materials

The following supporting information can be downloaded at: https://www.mdpi.com/article/10.3390/molecules29092145/s1, Figure S1: The effect of the LED light conditions on the content of identified CQAs of *R. carthamoides* shoots. Control—cool-white fluorescent light (FL); WL—white LED light; BL—blue LED light; RL—red LED light; R:BL—red and blue LED (70%:30%) light; agar—shoots grown on agar (0.7%) MS medium; liquid—shoots grown in liquid MS medium, in a 300 mL Erlenmeyer flask on a rotary shaker at 80 rpm; agar P1—shoots grown on agar (0.7%) MS medium directly exposed to LED lights; agar pre-P5—shoots grown on agar (0.7%) MS medium exposed to LED lights for 5 passages (35 days of each); Figure S2. The productivity of the sum of all identified CQAs (a) and flavonoids (b) (expressed in mg/L of medium) in *R. carthamoides* shoots cultured for 35 days in the liquid MS medium supplemented with IAA 0.1 mg/L and BA 0.5 mg/L in a 300 mL Erlenmeyer flask on a rotary shaker at 80 rpm. The results are means values ± SE. There is no difference (*p* < 0.05) among the means for the same metabolite marked with the same letter. Abbreviations refer to the conditions of the conducted analysis, as indicated below Figure S1; Figure S3. The effect of LED light conditions on the growth of *R. carthamoides* shoots. The results are means values ± SE. There is no difference (*p* < 0.05) among the means marked with the same letter. (a) % of buds compared to shoots; (b) hyperhydricity structures (%); (c) length of shoots (cm) and % of shoots of appropriate length. Abbreviations refer to the conditions of the conducted analysis, as indicated below Figure S1.
