# Supplementary material for: Light-Emitting Diodes and Liquid System Affect the Caffeoylquinic Acid Derivative and Flavonoid Production and Shoot Growth of Rhaponticum carthamoides (Willd.) Iljin
Source: Molecules. 2024 May 5;29(9):2145. doi: 10.3390/molecules29092145 (PMC11085107; doi:10.3390/molecules29092145)
Supplement: Supplementary file 1 [file molecules-29-02145-s001.zip › molecules-2915649-supplementary.pdf]

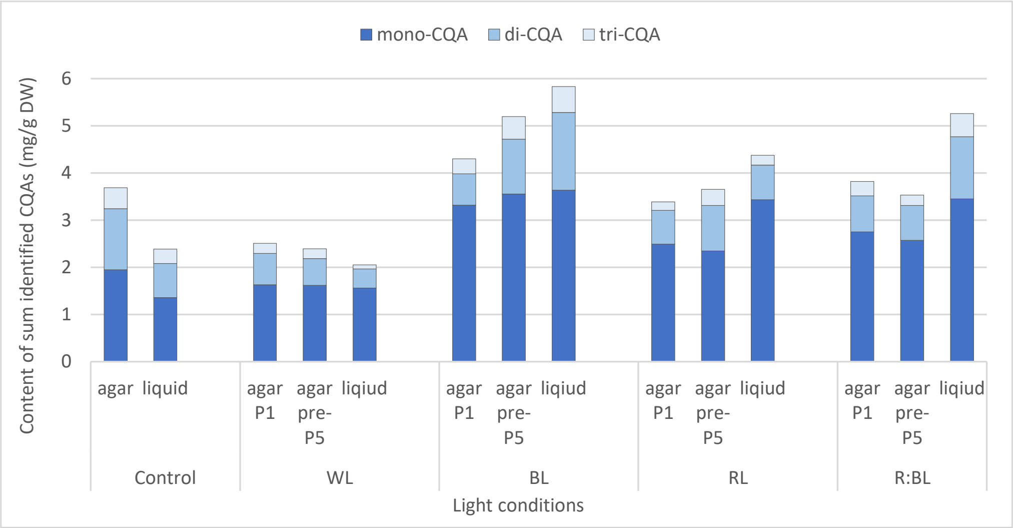

Figure S1. The effect of the LED light conditions on the content of identified CQAs of *R. carthamoides* shoots. **Control** – cool-white fluorescent light (FL); **WL** – white LED light; **BL** – blue LED light; **RL** – red LED light; **R:BL** – red and blue LED (70%:30%) light; agar – shoots grown on agar (0.7%) MS medium; **liquid** - shoots grown in liquid MS medium, in a 300 mL Erlenmeyer flask on a rotary shaker at 80 rpm; **agar P1** - shoots grown on agar (0.7%) MS medium directly exposed to LED lights; **agar pre-P5** - shoots grown on agar (0.7%) MS medium exposed to LED lights for 5 passages (35 days of each).

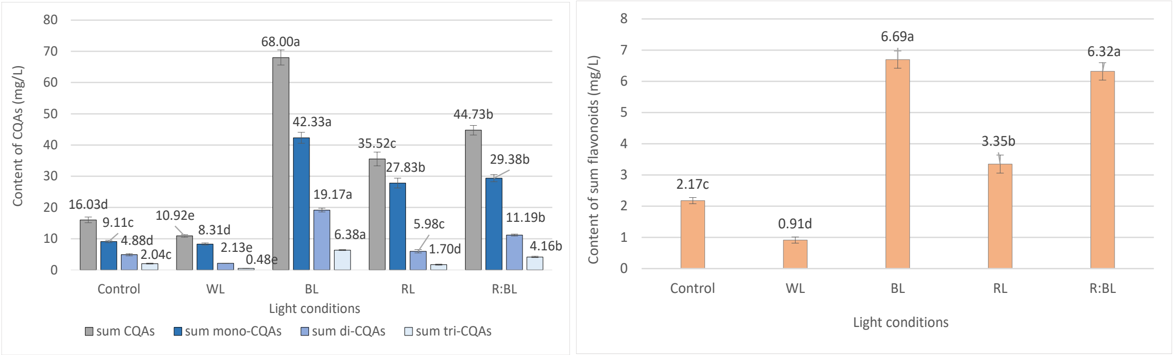

(a) **(b)**

Figure S2. The productivity of the sum of all identified CQAs (**a**) and flavonoids (**b**) (expressed in mg/L of medium) in *R. carthamoides* shoots cultured for 35 days in the liquid MS medium supplemented with IAA 0.1 mg/L and BA 0.5 mg/L in a 300 mL Erlenmeyer flask on a rotary shaker at 80 rpm. The results are means values ± SE. There is no difference ($p<0.05$) among the means for the same metabolite marked with the same letter. Abbreviations refer to the conditions of the conducted analysis, as indicated below Figure S1.

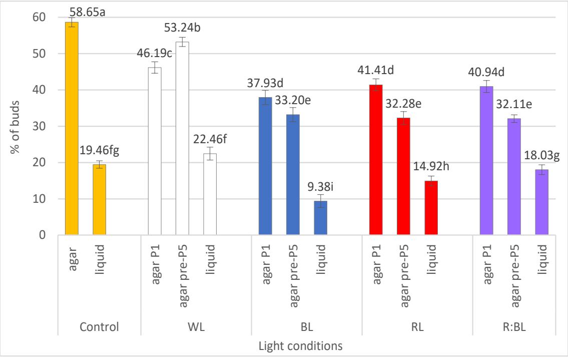

(a)

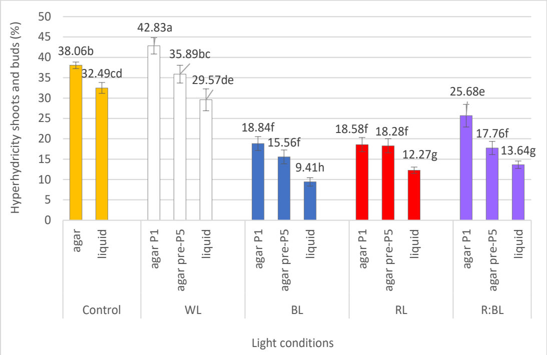

(b)

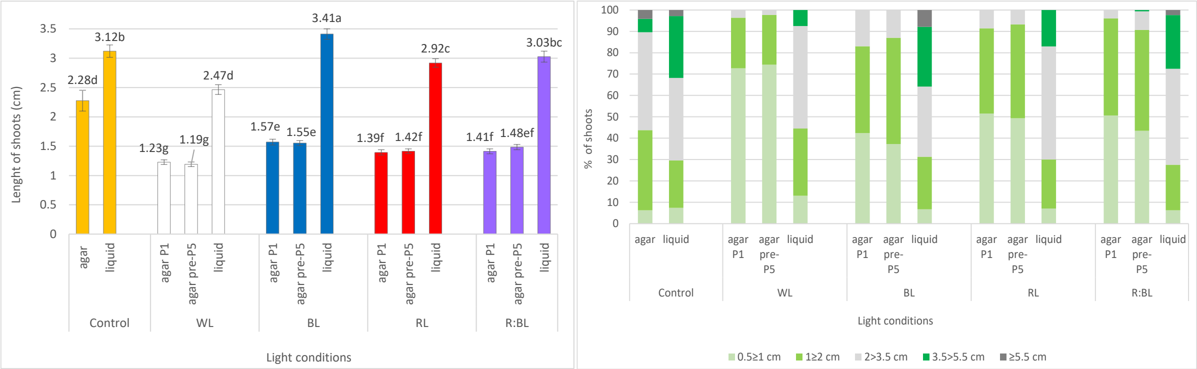

(c)

Figure S3. The effect of LED light conditions on the growth of *R. carthamoides* shoots. The results are means values ± SE. There is no difference ($p < 0.05$) among the means marked with the same letter. (**a**) % of buds compared to shoots; (**b**) hyperhydricity structures (%); (**c**) length of shoots (cm) and % of shoots of appropriate length. Abbreviations refer to the conditions of the conducted analysis, as indicated below Figure S1.
